# Paradoxical downregulation of LPAR3 exerts tumor-promoting activity through autophagy induction in Ras-transformed cells

**DOI:** 10.1186/s12885-022-10053-0

**Published:** 2022-09-10

**Authors:** Sung-Hee Hwang, Hye-Gyo Kim, Michael Lee

**Affiliations:** Division of Life Sciences, College of Life Sciences and Bioengineering, Incheon National University, 119 Academy-ro, Yeonsu-gu, Incheon, 22012 Republic of Korea

**Keywords:** LPAR3, Autophagy, Knockout, Cell transformation, Downregulation, Paradoxical, Methylation, Ras, Epigenetic

## Abstract

**Background:**

Lysophosphatidic acid receptor 3 (LPAR3) is coupled to G*α*_i/o_ and G*α*_11/q_ signaling. Previously, we reported that *LPAR3* is highly methylated in carcinogen-induced transformed cells. Here, we demonstrate that LPAR3 exhibits malignant transforming activities, despite being downregulated in transformed cells.

**Methods:**

The LPAR3 knockout (KO) in NIH 3 T3 and Bhas 42 cells was established using the CRISPR/Cas9 system. Both RT-PCR and DNA sequencing were performed to confirm the KO of LPAR3. The cellular effects of LPAR3 KO were further examined by WST-1 assay, immunoblotting analysis, transwell migration assay, colony formation assay, wound scratch assday, in vitro cell transformation assay, and autophagy assay.

**Results:**

In v-H-ras-transformed cells (Ras-NIH 3 T3) with LPAR3 downregulation, ectopic expression of LPAR3 significantly enhanced the migration. In particular, LPAR3 knockout (KO) in Bhas 42 (v-Ha-ras transfected Balb/c 3 T3) and NIH 3 T3 cells caused a decrease in cell survival, transformed foci, and colony formation. LPAR3 KO led to the robust accumulation of LC3-II and autophagosomes and inhibition of autophagic flux by disrupting autophagosome fusion with lysosome. Conversely, autolysosome maturation proceeded normally in Ras-NIH 3 T3 cells upon LPAR3 downregulation. Basal phosphorylation of MEK and ERK markedly increased in Ras-NIH 3 T3 cells, whereas being significantly lower in LPAR3 KO cells, suggesting that increased MEK signaling is involved in autophagosome–lysosome fusion in Ras-NIH 3 T3 cells.

**Conclusions:**

Paradoxical downregulation of LPAR3 exerts cooperative tumor-promoting activity with MEK activation through autophagy induction in Ras-transformed cells. Our findings have implications for the development of cancer chemotherapeutic approaches.

**Supplementary Information:**

The online version contains supplementary material available at 10.1186/s12885-022-10053-0.

## Background

Lysophosphatidic acid receptors (LPARs) are a group of heterotrimeric G protein-linked receptors (GPCRs) for lysophosphatidic acid (LPA) [1, 2]. At least six GPCRs are known to mediate the diverse effects of LPA on physiological, pathological, and developmental processes [3]. Previously, we found that among several LPAR subtypes, *LPAR3* was highly methylated in carcinogen-induced transformed Bhas 42 cells compared with untransformed cells [4]. LPAR3, which couples with G proteins, G*α*_i/o_ and G*α*_11/q_ [5], mediates Ras pathway and PI3K/Akt pathway, resulting in increased cell proliferation and suppression of apoptosis [1]. These results point to the functional redundancy of LPARs depending on the cellular context.

LPAR3 has been reported to regulate positively or negatively tumorigenesis [6]. In human ovarian cancer and rodent hepatoma cells, LPAR3 contributed to tumor-promoting activity [7–9]. Conversely, LPAR3 inhibited cell migration and invasion in human colorectal cancer and rat lung cancer [10, 11]. Thus, the role of LPAR3 in cancer remains unclear.

Furthermore, the expression patterns and levels of LPAR3 vary among malignancies [6]. The level of LPAR3 was upregulated in ovarian cancer cells compared with that in the corresponding normal tissue [12]. Conversely, LPAR3 expression is relatively lower in human and mouse cancer cells due to aberrant DNA methylation [13, 14]. Promoter hypermethylation leads to epigenetic gene silencing in cancer [15, 16]. We previously found that *LPAR3* was highly methylated in transformed foci of Bhas 42 cells compared with untransformed cells [4]. In this study, we generated LPAR3-knockout (KO) cells to assess the biological role of LPAR3 in tumorigenicity and cell migration ability. Our findings revealed that despite being downregulated in transformed cells, LPAR3 may act as a positive regulator in tumorigenesis. Moreover, LPAR3 downregulation might be associated with tumor progression concomitant with autophagy induction in Ras-transformed cells.

## Methods

### Cell lines and cell culture

Bhas 42 (v-Ha-ras transfected Balb/c 3 T3) cells were purchased from the Health Science Research Resources Bank (Osaka, Japan). Bhas 42 cells are regarded as a model for initiated cells in the cell transformation assay [17]. Bhas 42 cells were cultured in MEM (Thermo Fisher Scientific, Carlsbad, CA, USA) containing 10% FBS (M10F) at 37 °C under 5% CO_2_ and 95% O_2_, as described [4]. The v-Ha-ras-transformed NIH 3 T3 (Ras-NIH 3 T3) cells were previously described [18]. Ras-NIH 3 T3 and their parental cells were grown in DMEM containing 10% FBS. These cells were maintained in 75 cm^2^ vented flask until they reached a density of 70 ~ 80%.

### Immunofluorescence staining

Cells were cultured in 4-well chamber slides. Formalin-fixed cells were permeabilized for 15 min in 0.25% Triton X-100 (in DPBS), blocked, and incubated overnight with the anti-LPAR3 antibody (1:80, Abcam, Cambridge, MA, USA). Images were captured on Axio Imager Z1 fluorescence microscope (Carl Zeiss, Thornwood, NY, USA).

### Plasmid DNA, siRNA, and transient transfection

pCMV6-LPAR3 (Myc-DDK-tagged) (CAT# MR226015) was purchased from OriGene Technologies, Inc. (Rockville, MD, USA). pEGFP-LC3 (Addgene #11546), ptfLC3 (Addgene #21074) and pCI-neo-mAtg5 (Addgene #22956) were obtained from Addgene (Cambridge, MA, USA). Murine *Lpar3* siRNA was obtained from Integrated DNA Technologies (Coralville, IA, USA). The siRNA sequence was 5′-CUGAAAGGUAGAUCAGUUAAAAACA-3′ (sense). Cells were transiently transfected with the indicated constructs using Lipofectamine 2000.

### Measurement of autophagic flux by LC3 conversion

The relevant parameter in LC3 flux assay is the difference in the amount of LC3-II in the presence and absence of lysosomal inhibitors (chloroquine, CQ). Briefly, cells were treated with either vehicle or CQ (30 μM) for 24 h. Western blots were performed with antibody against LC3 as previously described [19]. Images were captured using the Bio-Rad ChemiDoc XRS+ instrument (Hercules, CA, USA). Band intensity values were measured using the Image Lab software, version 5.2.1 (Bio-Rad). LC3-II band intensity was normalized to that of a loading control, β-actin, and ratio further normalized to that in control (untreated). Autophagic flux was calculated by subtracting ratio untreated group from ratio of CQ-treated group.

### The tandem RFP-GFP-tagged LC3 fluorescence assay

Cells were transiently transfected with the tandem mRFP–GFP–LC3 reporter plasmid (ptfLC3). After 24 h, the cells were seeded on to 4-well chamber slide before fixation in 10% neutral-buffered formalin. Coverslips were mounted on slides using antifade mounting media. Twenty transfected cells were analyzed for each condition. Yellow puncta are indicators of autophagosomes, whereas red puncta are indicative of autolysosomes in merged image. The number of LC3 puncta was quantitatively assessed withe ImageJ software (NIH, Bethesda, MD, USA).

### Fusion of autophagosomes with lysosomes

Autophagosome fusion with lysosome was evaluated by analyzing the colocalization of fluorescent LC3-II with LysoTracker (a fluorescent acidotropic probe for lysosome labeling). For this experiment, cells were cultured in 4-well chamber slides and stained with 50 nM LysoTracker Red DND-99 (Thermo Fisher Scientific, Carlsbad, CA, USA). Formalin-fixed cells were permeabilized, and blocked for 1 h in 0.1% Tween 20 and 1% BSA.. The cells were then incubated overnight with anti-LC3 antibody followed by incubation with the FITC-labeled antibody for 60 min. Imaging was performed using the Axio Imager Z1 fluorescence microscope (Carl Zeiss).

### Generation of the LPAR3 KO cells with CRISPR/Cas9 system

The plasmid containing single guide RNA sequence designed against exon-2 of the *LPAR3* gene was obtained from ToolGen (Seoul, Korea). The sgRNA sequence is as follows: 5′-AAACGTTGACCGTCAACCGCTGG-3′. For the establishment of LPAR3 KO cell lines, pHRS_HumanLPAR3_CMV containing a hygromycin resistance gene (ToolGen) was used. This plasmid expresses a hygromycin resistance protein when the target sequences are cleaved by a nuclease. The knockout clones were generated as previously described [20]. Briefly, NIH 3 T3 cells were cultured in six-well dishes to 70–80% confluence. The cells were cotransfected with 1 μg of the *LPAR3* sgRNA plasmid, 1 μg of pRGEN-Cas9-CMV, and 1 μg of pHRS_HumanLPAR3_CMV using Lipofectamine 2000 (Life Technologies). After transfection, these cells were incubated with 150 μg/ml of hygromycin for 2 days. Surviving cells were reseeded at 0.4 cells/well of a 96-well plate for isolation of single-cell clones. The following primer sets were used for confirmation of genome editing by RT-PCR: 5′-ACCGTCAACCGCTGGT-3′ and 5′-CAATTCCATCCCAGCGTGG-3′. GAPDH was used as an internal control.

### In vitro two-stage cell-transformation assay

The in vitro two-stage cell transformation assay (CTA) was performed as described [21]. Briefly, cells were plated in 6-well plate at 4000 cells/well. At 24 h after initial culture, culture medium was replaced with medium containing 3-methylcholanthrene (MCA). Four days later, the culture medium was changed to fresh medium containing phorbol 12-myristate 13-acetate (PMA). The culture was continued in the medium containing PMA for 2 weeks, with media changes every 3–4 days. The cells were then cultured in DMEM supplemented with insulin-transferrin-ethanolamine-sodium selenite (ITES, Sigma, St. Louis, MO, USA) plus 2% FBS during the promotion period [22]. Five to six weeks after tumor initiator treatment, cells were stained with a 5% Giemsa solution.

### Cell survival assay

Cells were plated in 96-well microtiter plates as 5 × 10^3^ cells/well, and treated with appropriate drugs for 3 or 4 d. Then, the cells in each well were incubated with 10 μL of WST-1 (Takara Bio Inc., Otsu, Japan) for 1 h at 37 °C. The plates were read on a SpectraMax 190 reader (Molecular Devices, San Jose, CA, USA) at 450 nm.

### In vitro soft-agar colony-formation assay

To assess growth of transformed cells in soft agar, cells (1 × 10^4^) were seeded in triplicate at 1 × 10^4^ per 60-mm plate in culture medium containing 0.4% agarose over a base layer of culture medium containing 0.7% agarose followed by incubation for 3 weeks. Colonies were counted after staining with crystal violet.

### Transwell cell migration assay

Migration assay was performed in triplicate with the 24-well transwell with 8-μm pore filter (Millipore, Billerica, USA). Cells (5 × 10^4^ cells/well) were placed in DMEM medium without serum in the upper chamber, and lower camber was filled with medium containing 10% FBS. The cells were fixed, and stained with 0.05% crystal violet after additional culture for 8 h. The cells adhering to the underside of the filters were analyzed using light microscopy. Bound crystal violet was extracted with acetic acid, and quantified by measuring the absorbance at 550 nm.

### Wound scratch assay

Cell monolayers were wounded with a sterile 200 μL pipette tip. Images of the cells were acquired at 0, 4, 8, and 12 h after scratching with an inverted microscope (Zeiss Primo Vert) equipped with the Axiocam 105 camera and ZEN 2.6 software (Carl Zeiss Inc.). The TScratch software was used to quantify open surface areas [23].

### Immunoblot analysis

To prepare whole-cell lysates, cells were harvested by scraping into lysis buffer containing 1% Triton X-100 and protease inhibitors. Protein concentrations of cell lysates were determined using the Pierce BCA Protein Assay Kit (Thermo Fisher Scientific, Carlsbad, CA, USA). Western blots were performed as previously described [20]. Membranes containing phosphorylated proteins were immunoprobed with the corresponding antibodies: p-MEK, p-ERK, p-Akt, and p-mTOR (Cell Signaling Technology, Danvers, MA, USA). Images were captured using the Bio-Rad ChemiDoc XRS+ instrument (Hercules, CA, USA). Band intensity values were measured using the Image Lab software, version 5.2.1 (Bio-Rad).

### Quantitative real-time reverse transcription-PCR (qPCR) analysis

Total RNA extraction, cDNA synthesis, and qPCR were performed as previously described [24]. The primer sets (Bioneer, Daejeon, Korea) designed and used for qPCR analysis are listed in Supplementary Tables S1 and S2. The qPCR data were calculated using the 2^−ΔΔCt^ method [25] and normalized to GAPDH levels.

### Statistical analysis

Statistical analysis was performed using unpaired t test or by one-way ANOVA followed by Dunnett’s t test. Data are represented as the mean ± standard deviation of at least three independent experiments. The difference was considered significant if *p* value< 0.05. All analyses were performed using the GraphPad Prism software version 3.06 (GraphPad Software, CA, USA).

## Results

### LPAR3 downregulation in Ras-transformed cells

**Fig. 1:**
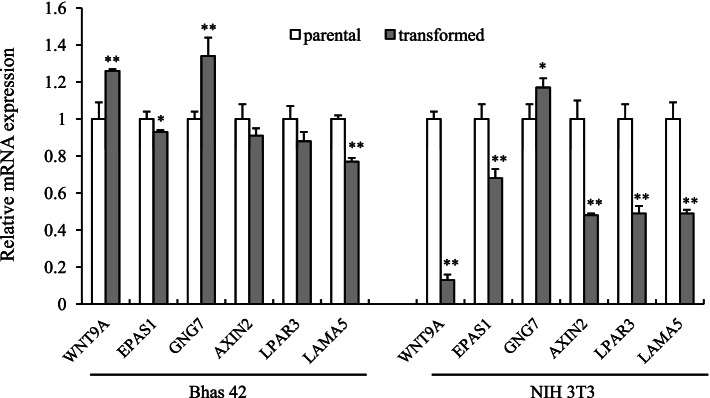
Relative gene expression of genes with hypermethylated DMRs in parental and their transformed cells. For quantitative analysis of gene expression, the comparative threshold cycle (Ct) Method for relative quantification (2^−ΔΔCt^) was used. The expression of the target genes was normalized to GAPDH expression. Values represent the mean ± SD of quadruplicate determinants from one of three representative experiments. **P < 0.01 and *P < 0.05 as determined by the unpaired t-test compared to parental cells

Promoter hypermethylation is an important epigenetic mechanism for inactivating cancer-related genes [26]. We previously found differentially methylated regions in 556 CpG dinucleotides in transformed foci of Bhas 42 cells [4]. In this study, we performed qPCR to confirm the downregulation of six genes, which belong to “pathways in cancer,” identified using functional enrichment analysis of differentially methylated regions. The expression of EPAS1, AXIN2, LPAR3, and LAMA5 was suppressed in association with cell transformation (Fig. 1). The downregulation of these four genes was confirmed in another transformed cell line, Ras-NIH 3 T3. LPAR3 is involved in cancer cell migration and invasion, although reports on LPAR3 expression in cancer are inconsistent [9, 11]. Therefore, we focused on LPAR3 and explored its role in carcinogenesis.

### LPAR3 as a candidate regulator of cell transformation

**Fig. 2:**
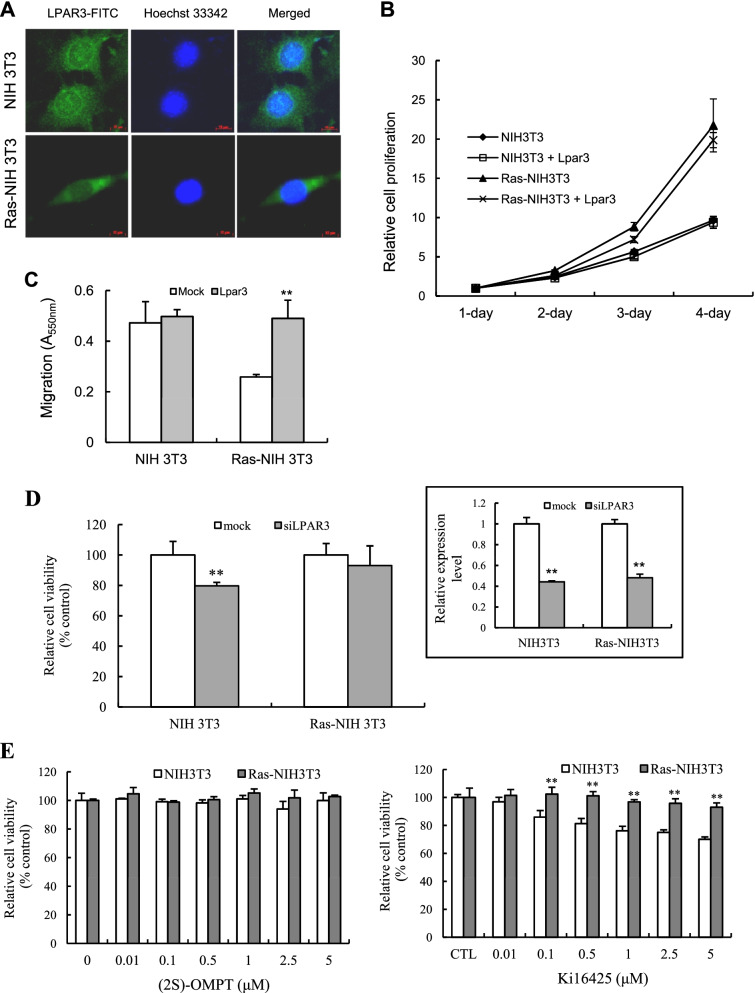
Effect of LPAR3 on cell survival and migration of parental and Ras-NIH 3 T3 cells. **A** Immunofluorescence analysis was performed to detect the endogenous expression of LPAR3 in NIH 3 T3 and Ras-NIH 3 T3 cells. Green and blue indicate LPAR3 expression and Hoechst 33342 nuclear staining, respectively. **B** Cell viability was evaluated in parental and Ras-NIH 3 T3 cells transiently transfected with LPAR3 for the indicated days. Results were expressed as the mean ± SD of quadruplicate determinations. **C** Transwell migration assays were performed with parental and Ras-NIH 3 T3 cells transiently transfected with LPAR3. Results were expressed as the mean ± SD of quadruplicate determinations. **D** Cell viability was determined in parental and Ras-NIH 3 T3 cells transiently transfected with *LPAR3* siRNA or a nontargeting control siRNA for 24 h, followed by incubation in 96-well plate for 3 days. Results were expressed as the mean ± SD of quadruplicate determinations. *LPAR3* knockdown was verified using qPCR (*right inset*). **E** Cell viability was determined in parental and Ras-NIH 3 T3 cells treated with or without (2S)-OMPT or Kil16425 for 72 h. Results were expressed as the mean ± SD of quadruplicate determinations. **P < 0.01 compared to NIH 3 T3 cells

To investigate whether LPAR3 expression contributes to cellular transformation, we first compared the endogenous levels of LPAR3 in Ras-NIH 3 T3 cells and their parental cells (Fig. 2A). Consistent with the results shown in Fig. 1, results from immunofluorescence staining revealed that LPAR3 expression was significantly lower in Ras-NIH 3 T3 cells than in their parental NIH 3 T3 cells. In parental cells, LPAR3 was expressed in the cytoplasm, especially in peripnuclear regions, in the distinctive punctuate pattern. In Ras-NIH 3 T3 cells, the LPAR3 expression was more homogeneous and diffuse. Next, we investigated the effects of LPAR3 overexpression on cell survival and migration. LPAR3 overexpression did not significantly affect the survival of both Ras-NIH 3 T3 and parental cells compared with that of mock-transfected cells (Fig. 2B). However, results from the transwell assay showed that ectopic expression of LPAR3 significantly enhanced the migration of Ras-NIH 3 T3 cells, but had little or no effect on parental cells (Fig. 2C). The migration of Ras-NIH 3 T3 cells with downregulated LPAR3 was much lower than that of NIH 3 T3 cells. On the other hand, LPAR3 knockdown with siRNA significantly lowered the viability of NIH 3 T3 cells (Fig. 2D). Moreover, treatment with LPAR3 antagonist Ki16425, inhibited the survival of parental NIH 3 T3 cells but did not affect the survival of Ras-NIH 3 T3 cells (Fig. 2E). A selective agonist of LPAR3, (2S)-OMPT, had no effect on the survival of two cell lines regardless of their transformation status. These findings suggest that, despite being downregulated in transformed cells, LPAR3 plays a positive role in migration and cell survival.

### Generation of LPAR3 knockout cell lines

**Fig. 3:**
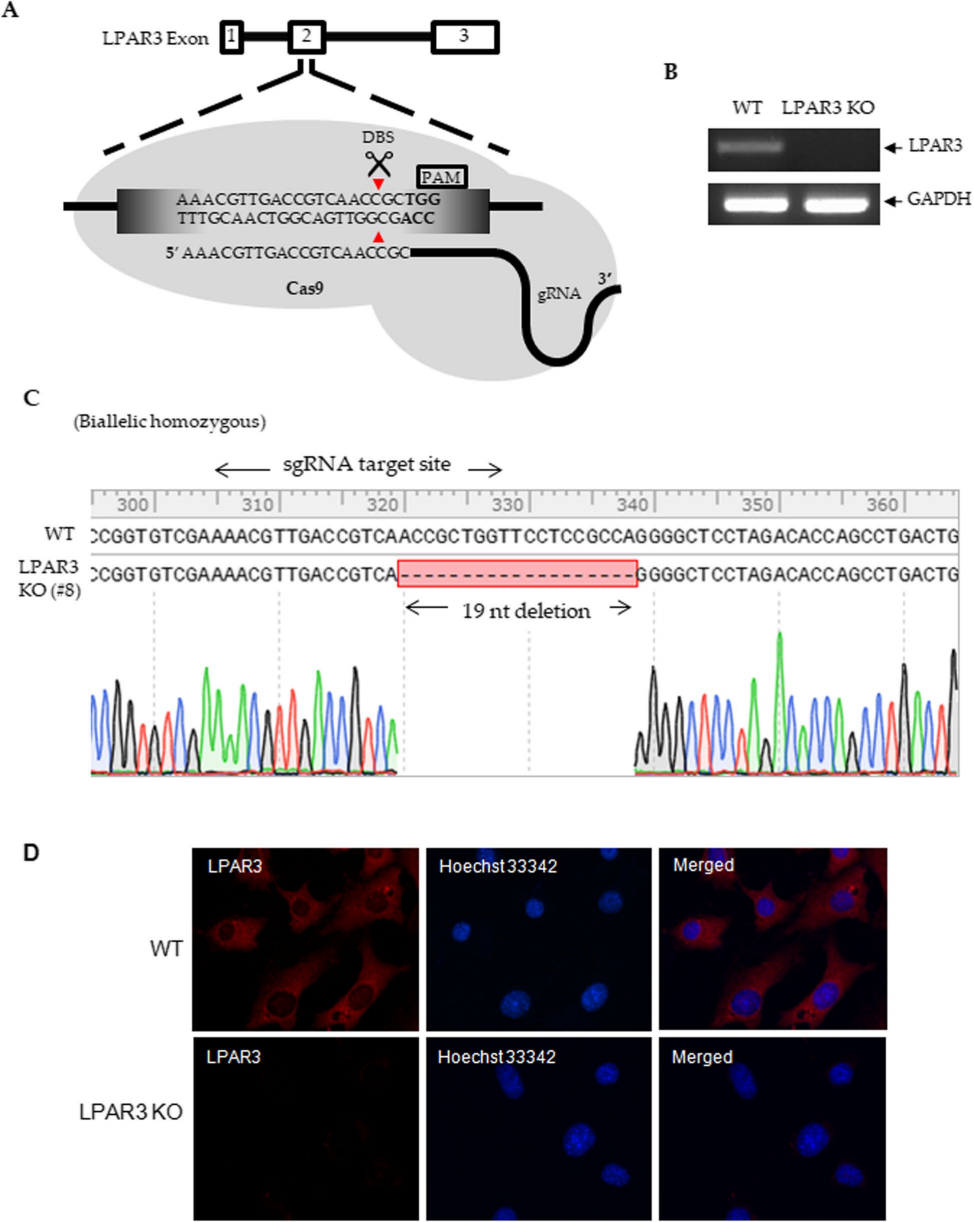
Generation of the LPAR3 knockout in NIH 3 T3 cells. **A** *LPAR3* sg RNA and target site were shown. **B**, **C** Generation of *LPAR3* KO was confirmed by RT-PCR (**B**) and sequencing target site (**C**). **D** Representative immunofluorescence images of LPAR3 KO and its parental NIH 3 T3 cells stained with Hoechst 33342 (nuclear stain [blue]) or antibodies selective against LPAR3 (red). DSB, double strand breaks; PAM, protospacer adjacent motif

To elucidate the role of LPAR3 in tumorigenesis, we established LPAR3 KO lines using CRISPR/Cas9. Figure 3A shows the *LPAR3* sg RNA and target site. Sequencing confirmed LPAR3 gene editing at the target site in four of nine initial clones in NIH 3 T3 cells. One clone (clone 8) with homozygous editions was selected for functional analysis (Fig. 3C). LPAR3 knockout of clone 8 was confirmed using RT-PCR (Fig. 3B). Immunofluorescence analysis revealed strong LPAR3 expression in the cytoplasm and cell membrane of parental cells and not LPAR3 KO cells (Fig. 3D). In addition, we generated knockout cell lines for LPAR3 in Bhas 42 cells. One clone with biallelic heterozygous editions was selected for functional analysis (Supplementary Fig. S1).

### LPAR3 knockout suppresses cell survival and migration in NIH 3 T3 and Bhas 42 cells

**Fig. 4:**
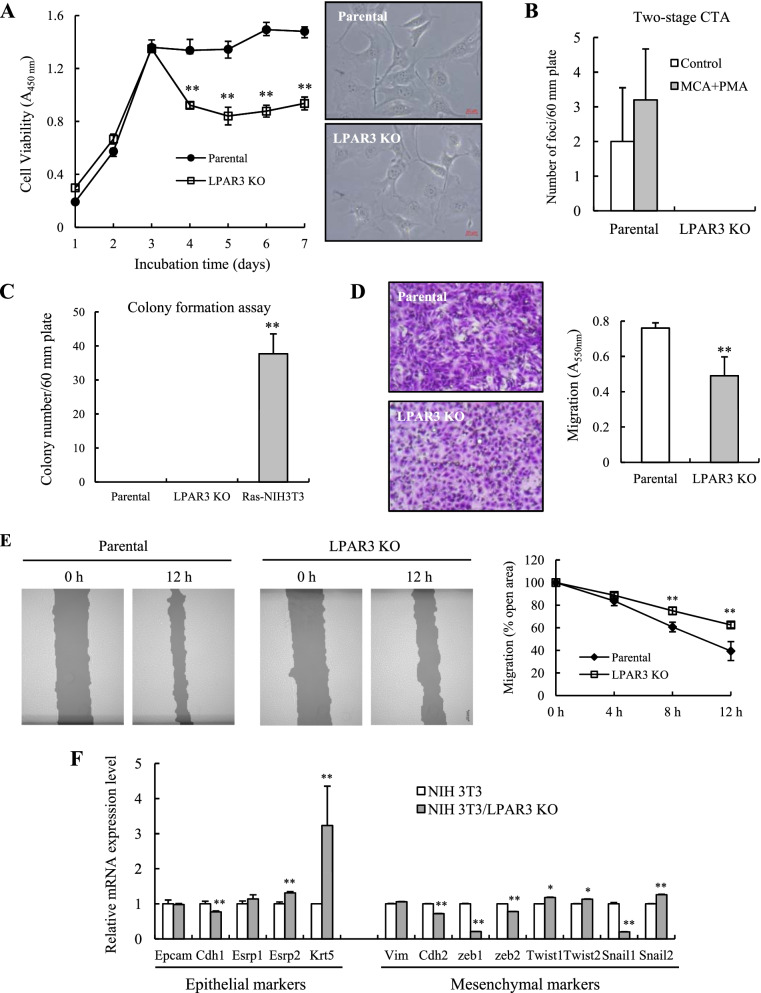
LPAR3 knockout inhibits cell survival and migration of NIH 3 T3 cells. **A** Cell viability was evaluated in parental and LPAR3 KO NIH 3 T3 cells incubated without any treatment for the indicated days. Results were expressed as the mean ± SD of quadruplicate determinations. In the *right inset*, pictures illustrate morphological changes in NIH 3 T3 cells after LPAR3 knockout. **B** In vitro two-stage CTA was performed with NIH/3 T3 cells using MCA as tumor initiator and PMA as tumor promotor, respectively. Cells were Giemsa stained to visualize malignant foci. Transformation frequency was expressed as the mean ± SD of sextuplicate determinations. **C** For anchorage-independent colony-formation assay, cells were grown in soft agar for 3 weeks. Results were expressed as the mean numbers of colonies per plate. Assays were performed in triplicate. **D** Transwell migration assays were performed with parental NIH 3 T3 and LPAR3 KO cells. Results were expressed as the mean ± SD of quadruplicate determinations. Representative images were shown in left panel. **E** A wound scratch assay was performed with parental NIH 3 T3 and LPAR3 KO cells. Representative images of scratch wound closure were photographed right and 12 h after the scratch (*left panel*). The TScratch software was used for determining the size of cell-covered areas (*right panel*). **F** RT-qPCR analysis was performed to quantify mRNA expression levels of epithelial and mesenchymal markers from both parental and LPAR3 KO NIH 3 T3 cells. The qPCR results was analyzed using the comparative threshold cycle (Ct) method. Results were normalized to that of GAPDH as a reference gene. Each point represents mean ± SD of quadruplicate determinations. *P < 0.05; **P < 0.01 compared to parental NIH 3 T3 cells

**Fig. 5:**
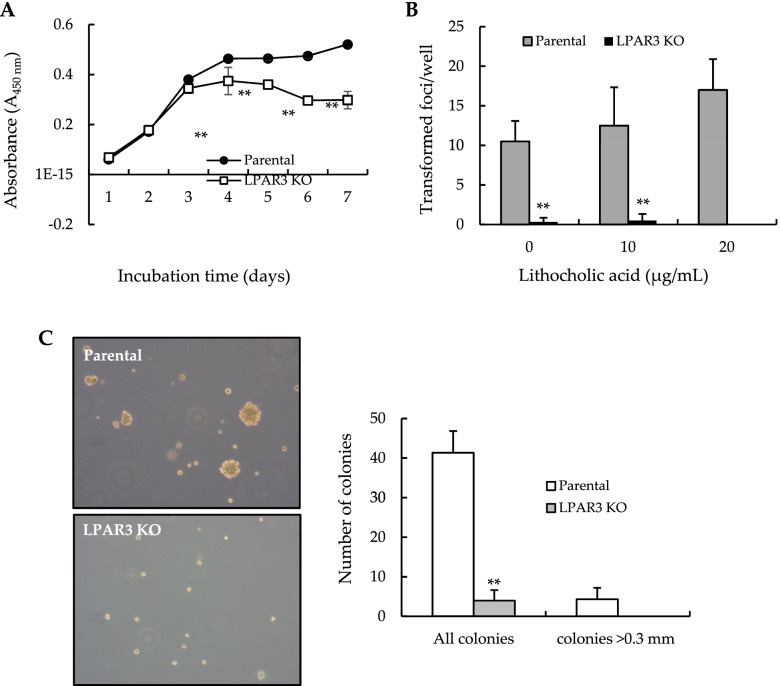
The effect of LPAR3 KO on the survival and migration of Bhas 42 cells. **A** Cell viability was determined in parental and LPAR3 KO Bhas 42 cells without any treatment for the indicated days. Data was expressed as the mean ± SD of quadruplicate determinations. **B** Parental and LPAR3 KO Bhas 42 cells were cultured in the presence of lithocholic acid as tumor promotor (days 4–14). After 21 d, the cells were fixed, and Giemsa-stained to visualize malignant foci. Transformation frequency was expressed as the mean ± SD of sextuplicate determinations. **C** For anchorage-independent colony-formation assay, cells were grown in soft agar for 3 weeks. **A** representative photograph showing anchorage-dependent colony formation by parental and LPAR3 KO Bhas 42 cells. The *right panel* shows the average numbers of colonies per plate. Assays were performed in triplicate. All the colonies or colonies > 0.3-mm in size in each well were counted under a microscope (× 10 magnification). **P < 0.01 compared to parental NIH 3 T3 cells

We conducted cell-survival, −migration, and -transformation assays using LPAR3 KO NIH 3 T3 cells. As shown in Fig. 4A, the survival of parental cells reached the maximum level on day 3 and remained at this level for 7 d. However, the survival of LPAR3 KO cells was maximum on day 3 and declined thereafter. In addition, LPAR3 knockout led to a partial morphological change in NIH-3 T3 cells, characterized by the appearance of shorter cytoplasmic extensions (Fig. 4A, *right inset*). These results suggest that LPAR3 signaling pathways contribute to cell survival and morphological changes. Findings from the in vitro two-stage CTA revealed that LPAR3 KO did not cause malignant transformation of in NIH 3 T3 cells (Fig. 4B). Results from the soft agar colony-formation assay revealed that almost no clones of LPAR3 KO and its parental NIH3T3 cells, but many colonies of Ras-NIH 3 T3 cells, were formed (Fig. 4C). Results from the transwell and wound scratch assays revealed that LPAR3 KO significantly reduced NIH 3 T3 cell migration (Fig. 4D and E). Because epithelial–mesenchymal transition (EMT) contributes to cancer progression [27], we quantitatively examined mesenchymal/epithelial markers in parental and LPAR3 KO NIH 3 T3 cells (Fig. 4F). Epithelial markers, including Krt5 and Esrp2, were expressed at higher levels, whereas mesenchymal markers, including Cdh2, Zeb1, and Snail1, were expressed at lower levels in LPAR3 KO than parental cells. Consistent with results for LPAR3 KO in NIH 3 T3 cells, LPAR3 KO suppressed the survival of Bhas 42 cells (Fig. 5A). Moreover, LPAR3 loss in Bhas 42 cells almost completely inhibited transformation by NGTxC, lithocholic acid (Fig. 5B), and reduced colony formation on soft agar (Fig. 5C). Thus, LPAR3 may confer malignant properties on Ras-transformed cells, such as Ras-NIH 3 T3 and Bhas 42 cells.

### LPAR3 mediates survival and migration of NIH 3 T3 cells through the G_i/o_–MAPK pathway

**Fig. 6:**
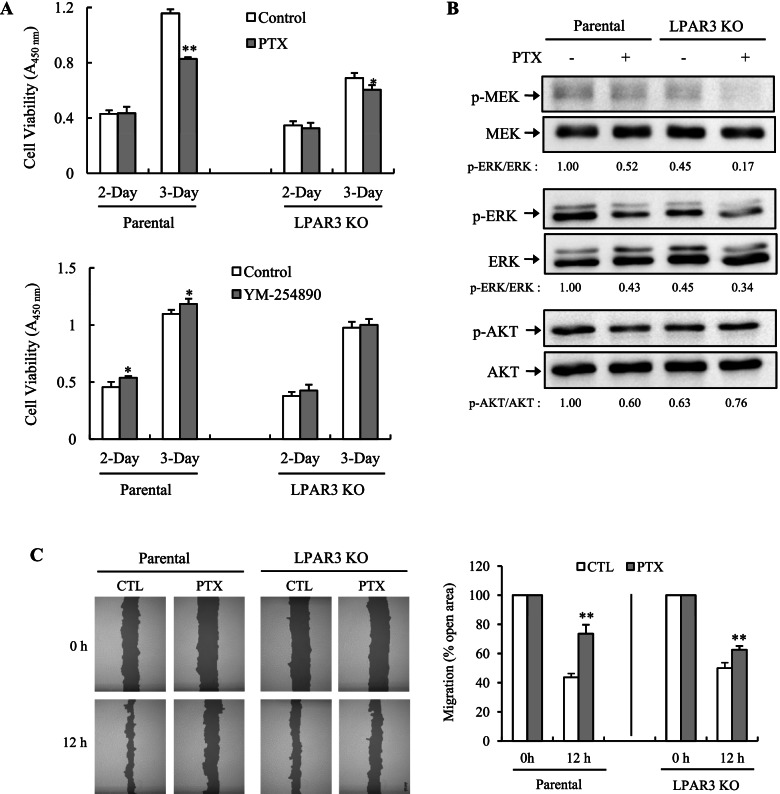
Involvement of the Gi pathway in LPAR3-mediated survival and migration of NIH 3 T3 cells. **A** Cell viability was evaluated in parental and LPAR3 KO NIH 3 T3 cells treated with PTX (100 ng/mL) or YM-254890 (10 μM). Viability was expressed as the mean ± SD of quadruplicates. **B** Phosphorylated MEK, ERK, and AKT were detected through immunoblotting in parental and LPAR3 KO NIH 3 T3 cells treated with PTX for 48 h. The indicated values below each band represent normalized ratio of phosphorylated protein to total protein. **C** A wound scratch assay was performed with parental NIH 3 T3 and LPAR3 KO cells treated with or without PTX. Representative images of scratch wound closure were photographed right and 12 h after the scratch (*left panel*). Photographs shown in left panel was analyzed using the TScratch software for determining the size of cell-covered areas (*right panel*). *P < 0.05; **P < 0.01 compared to control

LPAR3 was reported to be coupled to G_i/o_ and G_q/11_ [28]. We examined which G protein is involved in LPAR3-mediated survival and migration. A G_i/o_-specific inhibitor, PTX, markedly inhibited the survival of NIH 3 T3 cells, whereas a G_αq/11_-specific inhibitor, YM-254890, exerted little effect on NIH 3 T3 cell survival (Fig. 6A). Both PTX and YM-254890 exhibited little or no suppressive effect on the survival of LPAR3 KO cells. By contrast, p-MEK/p-ERK levels of LPAR3 KO cells were lower than those of parental cells (Fig. 6B). This result is in agreement with other reports indicating that LPAR3 induces MAPK activation by coupling with Gα_i/o_ [28]. Levels of Akt phosphorylation were lower in LPAR3 KO cells than parental cells. PTX treatment reduced p-MEK/−ERK levels but had no significant effect on p-AKT levels in either cell line. PTX-induced inhibitory effects on p-MEK levels in LPAR3 KO cells suggest that LPARs are functionally redundant. Next, to find out the effects of PTX on migration, we performed a wound scratch assay in parental and LPAR3 KO cells (Fig. 6C). We observed wound closure in both cells after serum-deprivation for 12 h. Parental cells occupied significantly more surface area 12 h after wounding than LPAR3 KO cells. PTX had a marked suppressive effect on cell migration in both cell lines. Thus, these results imply that LPAR3 mediates the signaling pathway for cell survival and migration through the PTX-sensitive G_i/o_–MAPK pathway-dependent mechanism in NIH 3 T3 cells.

### Downregulation of LPAR3 increases autophagic flux in Ras-transformed cells

**Fig. 7:**
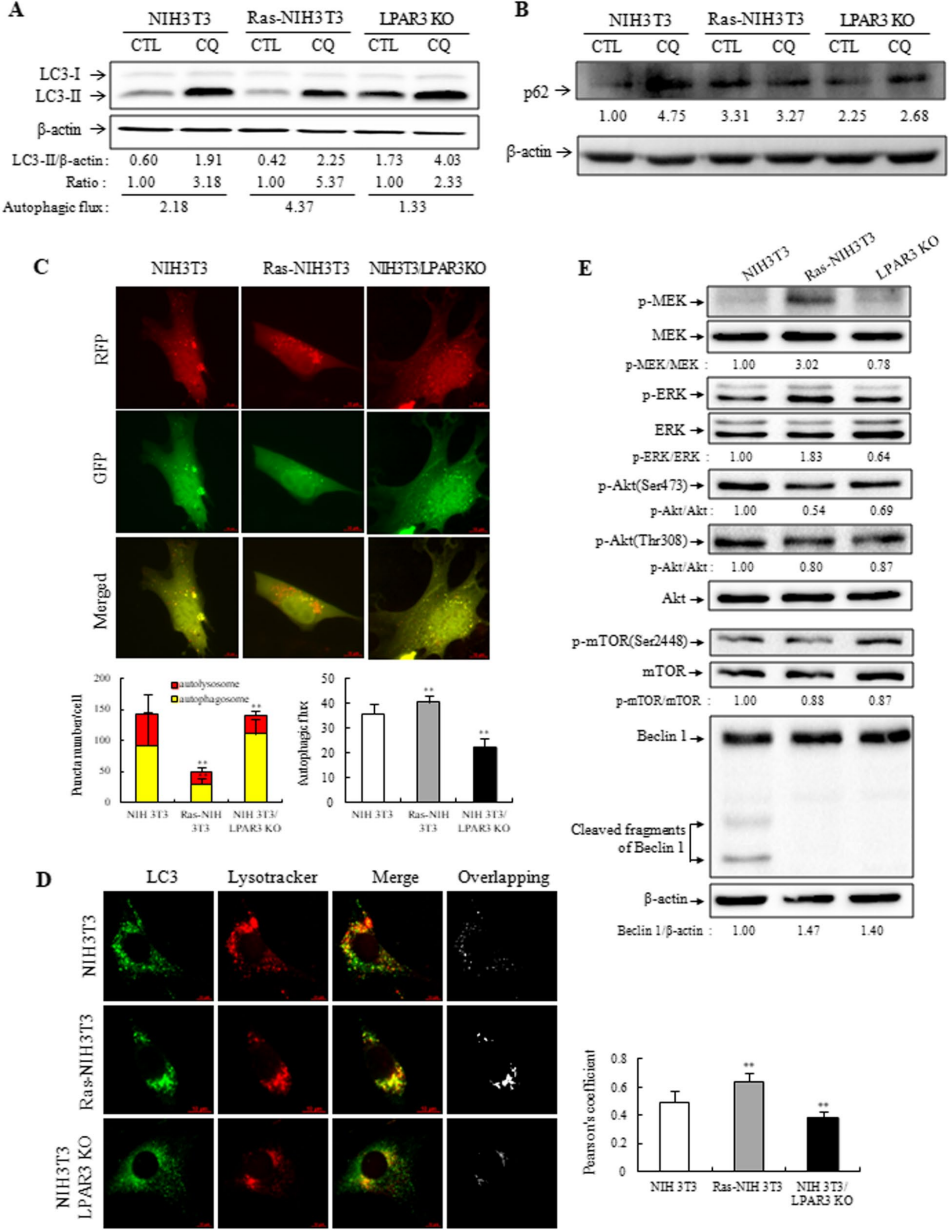
Increase in autophagic flux in Ras-NIH 3 T3 cells. **A** Immunoblotting was performed to measure LC3 conversion in cells treated with or without 30 μM CQ for 24 h. The values below each band represent autophagic flux as the difference in the amount of LC3-II normalized to loading control, β-actin, between the CQ-treated and untreated groups. **B** Protein expression levels of p62 were measured by immunoblot analysis. Representative images are presented along with β-actin as an internal loading control. The indicated values below each band represent normalized ratio of phosphorylated protein to total protein. The p62 protein level was normalized to β-actin and was shown as relative value below each band. The intensity value observed in control of NIH 3 T3 cells was defined as 1.0. **C** Autophagic flux was determined using ptfLC3 imaging assays. Representative images are shown in the upper panel. Yellow puncta are indicators of autophagosomes, whereas red puncta are indicative of autolysosomes in merged image. In *lower panel*, the average numbers of yellow and red puncta/cell were shown as a bar graph. Data are expressed as mean ± SD of at least 20 different cells. **D** Colocalization analysis of LC3 and Lysotracker. The cells were incubated in serum-free media containing 50 nM Lysotracker (red) stained with Lysotracker (red). Endogenous LC3 was visualized with the anti-LC3 antibody (green). Overlapping analysis was performed using the RG2B Colocalization plugin in ImageJ. Bar, 10 μm. *Lower panel*: LC3 puncta that colocalized with Lysotracker were counted in 50 cells using the JACoP plugin in ImageJ. The data shown are the average and standard deviation of ten randomly selected images. **P < 0.01 compared to NIH 3 T3 cells. **E** The phosphorylation of MEK, ERK, Akt, and mTOR was detected through immunoblotting. The indicated values below each band represent normalized ratio of phosphorylated protein to total protein

Autophagy functions as a prosurvival or proapoptotic process in tumorigenesis [29]. LPA inhibits autophagy by activating LPAR3 [30]. Therefore, we investigated the link between LPAR3 expression and basal autophagy in cells expressing different levels of LPAR3, namely parental, LPAR3 KO, and Ras-NIH 3 T3 cells. First, basal autophagic flux was evaluated in cells treated with the late-stage autophagy inhibitor CQ. Autophagic flux was estimated as the differential amount of LC3-II normalized to loading control β-actin [31]. Autophagic flux was higher in Ras-NIH 3 T3 cells and lower in LPAR3 KO cells than in NIH 3 T3 cells (Fig. 7A). Interestingly, LC3-II formation was greatly reduced in Ras-NIH 3 T3 cells, but increased in LPAR3 KO cells. We further performed immunoblot analysis for another autophagy marker p62 (Fig. 7B). We found a significant accumulation of p62 regardless of CQ treatment in NIH 3 T3 LPAR3 KO cells, in which autophagic flux was low, resulting in autophagosome accumulation. As expected, CQ treatment of NIH 3 T3 cells resulted in accumulation of p62 protein. Interestingly, despite high autophagic flux, Ras-NIH 3 T3 cells showed a high level of p62 protein, which were independent of CQ treatment. In particular, p62 has been known to be required for efficient tumorigenesis by Ras [32]. Also, p62 expression was reported to be upregulated in several malignant cells [33]. To provide more evidence that LPAR3 knockout blocks autophagic flux in NIH 3 T3 cells, we measured autophagic flux using ptfLC3 imaging assays (Fig. 7C). Although knocking out *LPAR3* did not affect autophagosome formation, a lower number of autolysosomes was observed in LPAR3 KO cells than parental cells. These results suggest that LPAR3 KO impairs autophagosome fusion with lysosome, followed by autophagosome accumulation in the cytosol. Thus, to elucidate whether LPAR3 knockout inhibits the fusion process, we analyzed the colocalization of endogenous LC3 foci with lysosomes (Fig. 7D). This process was monitored by analyzing the colocalization of fluorescent LC3-II and Lysotracker. We found that LPAR3 knockout in NIH 3 T3 cells causes autophagic flux inhibition by preventing autophagosome–lysosome fusion. Interestingly, despite the downregulation of LPAR3, autolysosome maturation proceeded normally in Ras-NIH 3 T3 cells. This difference between LPAR3 KO cells and Ras-NIH 3 T3 cells may be due to the signaling pathway activated by oncogenic Ras. The expression of activated oncogenic Ras increases basal autophagy, which can promote tumor growth [32, 34]. Because Ras activation is a major trigger for signaling cascades that activate the PI-3 kinase/AKT pathway and RAF pathway [35], immunoblotting was performed to assess the activation state of Akt/mTOR and MEK/ERK in the three cell lines (Fig. 7E). Levels of p-AKT in Ras-NIH 3 T3 and LPAR3 KO cells were lower than those in their parental cells, although levels of downstream effectors of AKT and p-mTOR were not substantially different from those of parental cells. Although Ras-NIH 3 T3 and LPAR3 KO cells showed similar levels of inhibition in the AKT/mTOR pathway, they showed completely opposite activation states in the MEK/ERK pathway. Basal phosphorylation of MEK and ERK increased significantly in Ras-NIH 3 T3 cells, whereas in LPAR3 KO cells, it decreased, compared with parental cells, implying that LPAR3 KO inhibits autophagy by suppressing the MEK/ERK signaling pathway and not the mTOR-dependent pathway. Interestingly, we found several fragments of Beclin 1 in parental cells, but not in both LPAR3 KO and Ras-NIH 3 T3 cells, in which LPAR3 expression was lost or significantly reduced. Thus, cooperation between LPAR3 downregulation and MEK activation is likely required for autophagy induction in Ras-NIH 3 T3 cells.

## Discussion

Promoter hypermethylation is one of the main epigenetic mechanisms for inactivating tumor-promoting genes [15, 26]. We previously found that *LPAR3* is highly methylated in carcinogen-induced, transformed Bhas 42 cells compared with untransformed cells [4]. In this study, we found that high levels of CpG methylation in *LPAR3* in transformed Bhas 42 cells were associated with the downregulation of LPAR3. The transformation-associated downregulation of LPAR3 was also confirmed in Ras-NIH 3 T3 cells, which showed transformed morphology and grew colonies on soft agar [18]. These results indicate that LPAR3 expression was generally downregulated in malignant transformed cells. Consistent with our results, several studies have suggested that LPAR3 is epigenetically silenced in tumor cells through the hypermethylation of its promoter [13, 14]. However, data on LPAR3 expression in cancer are inconsistent; LPAR3 expression is elevated in ovarian cancer cells [36]. These results suggest that LPAR3 expression levels depend on cancer cell type.

Recent reports have demonstrated that LPAR3 regulates either positively or negatively cancer cell progression depending on tumor cell type [9], implying a more complicated role for LPAR3. In this study, the downregulation of LPAR3 during cellular transformation suggests that LPAR3 might act as a tumor suppressor in tumorigenesis. Although several studies have reported a suppressive role of LPAR3 in carcinogenesis [10, 11], contradictory reports on LPAR3 promoting cancer progression also exist [6, 9, 37, 38]. Here, we found that LPAR3 expression positively correlated with migration in NIH 3 T3 cells despite the downregulation of LPAR3 during cellular transformation. In particular, NIH 3 T3 cells lacking LPAR3, knocked out with CRISPR/Cas9-mediated genome editing, exhibited significantly lower cell motility than parental cells and lost susceptibility to cellular transformation. In addition, the survival of LPAR3 KO cells was maximum on day 3 and declined thereafter, while the survival of parental cells remained at this level for 7 d. These findings are well consistent with the previous report that LPAR3 acts as a major promoter of long-term viability among malignant tumor cells [39]. Moreover, mesenchymal markers, including Cdh2, Zeb1, and Snail1, were expressed at lower levels in LPAR3 KO cells than in parental cells. Conversely, LPAR3 KO cells expressed the epithelial marker Krt5. These findings suggest a role for LPAR3 in the initial stages of metastasis, when the EMT program starts. Thus, LPAR3 may act as a positive regulator of malignant properties in transformed cells.

Autophagy plays a dual role in tumor progression [40]. Previously, we showed that malignant transformation was accelerated in murine cell line models with defects in autophagy caused by the knockout of ATG5 [20], implying a protective role of autophagy against tumorigenesis. Although the role of LPAR3 in tumorigenesis has been extensively studied, few studies have linked LPAR3 activity to autophagy. In particular, Yang et al. [29] suggested that LPA inhibits autophagy via the LPAR3/AKT/mTOR Pathway. Here, we found that LPAR3 KO in NIH 3 T3 cells caused a robust accumulation of LC3-II and autophagosomes, resulting in a decrease in autophagic flux. In particular, LPAR3 knockout significantly reduced colocalization between LC3 and Lysotracker, indicating that LPAR3 loss inhibits the autophagy maturation process by impairing the fusion of autophagosomes and lysosomes. Cell viability assays revealed that LPAR3 knockout-induced autophagosome accumulation was associated with a decline in cell survival in LPAR3 KO cells. Increased autophagosome accumulation may be a contributing factor to toxicity caused by defects in autophagosome–lysosome fusion [41]. By contrast, LC3-II formation was greatly reduced in Ras-NIH 3 T3 cells, concomitant with a marked increase in autophagic flux. Overall reduced LC3-II (and autophagosomes) may reflect either reduced autophagosome initiation or enhanced autolysosome formation. Ras-NIH 3 T3 cells showed a large enhancement in LC3-II in the presence of CQ, suggesting an increase in autophagic flux from autophagosomes to autolysosomes.

Because autophagic flux significantly decreased in LPAR3 KO cells, LPAR3 signaling may act as an autophagy activator rather than an inhibitor in tumor cells. However, our findings also indicate that downregulation of LPAR3 due to promoter methylation enhances tumor progression by increasing autophagic flux in Ras-transformed cells. Consistently, autophagic flux was increased in Ras-NIH 3 T3 cells, which exhibited the downregulation of LPAR3. A plausible explanation for the discrepancy between the two cell lines is the activation state of signal transduction pathways leading to autophagy regulation between transformed (Ras-NIH 3 T3) and parental NIH 3 T3 cells. Several types of Ras-activated tumors have a high basal level of autophagy [42]. LPAR3 is coupled mainly with Ras/RAF/MAPK and PI3K/AKT/mTOR pathways [43, 44]. Although the two cell lines (Ras-NIH 3 T3 and LPAR3 KO) exhibited no substantial difference in phospho-mTOR levels from parental cells, they showed completely opposite activation states in the MEK and ERK pathways. Basal phosphorylation of MEK and ERK was largely enhanced in Ras-NIH 3 T3 cells, whereas it was significantly downregulated in LPAR3 KO cells. Our findings suggest that the ability to increase autophagic flux in Ras-transformed cells was partially regulated by an increase in MEK/ERK activity. MEK/ERK activation increases autophagosome and lysosome fusion, thereby increasing autophagic flux [45]. By contrast, we found several fragments of Beclin 1 in parental cells but not in LPAR3 KO and Ras-NIH 3 T3 cells, in which LPAR3 expression was lost or significantly reduced. It has been proposed that Beclin-1 cleavage by caspase inactivates Beclin-1-induced autophagy [46, 47]. Thus, we cannot exclude the possibility that an increase in autophagic flux is associated with the appearance of Beclin 1 fragments in Ras-transformed cells. Similar to Ras-transformed cells, Beclin 1 was not cleaved in LPAR3 KO cells; however, we speculate that autophagic flux decreased due to decreased MEK activity. These results suggest that cooperation between LPAR3 downregulation and MEK activation is required for autophagy induction in Ras-transformed cells. Although the direct coupling of LPAR3 to Ras/Raf/MEK/ERK to increase autophagic flux has not been demonstrated, our data have expanded our understanding of LPAR3 signaling.

## Conclusion

Our results suggest that the downregulation of LPAR3 exerts cooperative tumor-promoting activity with MEK activation through autophagy induction in Ras-transformed cells. These findings on the role of LPAR3 in cellular transformation have possible implications for new therapeutic and chemopreventive approaches. To better understand the biological significance of LPAR3 in tumor cells, we are investigating the changes in LPAR3 expression and autophagic flux during cellular transformation using an in vitro two-stage CTA model system.

## Supplementary Information

**Additional file 1: Suppl. Fig. S1.** Generation and validation of the knockout of LPAR3 in Bhas 42 cells LPAR3 KO was confirmed by sequencing for genomic editing at the target site.

**Additional file 2: Supplementary Table S1.** Real-time PCR primers sequence for genes with hypermethylated DMRs.

**Additional file 3: Supplementary Table S2.** Real-time PCR primers sequence for genes associated with EMT.

**Additional file 4.**

## Data Availability

All data generated or analyzed during this study are included in this published article and its Supplementary information files.

## References

[1] Lin ME, Herr DR, Chun J (2010). Lysophosphatidic acid (LPA) receptors: signaling properties and disease relevance. Prostaglandins Other Lipid Mediat.

[2] Ishii I, Fukushima N, Ye X, Chun J (2004). Lysophospholipid receptors: signaling and biology. Annu Rev Biochem.

[3] Yung YC, Stoddard NC, Chun J (2014). LPA receptor signaling: pharmacology, physiology, and pathophysiology. J Lipid Res.

[4] Hwang SH, Yeom H, Eom SY, Lee YM, Lee M (2019). Genome-wide DNA methylation changes in transformed foci induced by nongenotoxic carcinogens. Environ Mol Mutagen.

[5] Hama K, Aoki J (2010). LPA(3), a unique G protein-coupled receptor for lysophosphatidic acid. Prog Lipid Res.

[6] Tanabe E, Kitayoshi M, Yoshikawa K, Shibata A, Honoki K, Fukushima N (2012). Loss of lysophosphatidic acid receptor-3 suppresses cell migration activity of human sarcoma cells. J Recept Signal Transduct Res.

[7] Yu S, Murph MM, Lu Y, Liu S, Hall HS, Liu J (2008). Lysophosphatidic acid receptors determine tumorigenicity and aggressiveness of ovarian cancer cells. J Natl Cancer Inst.

[8] Cai H, Xu Y (2013). The role of LPA and YAP signaling in long-term migration of human ovarian cancer cells. Cell Commun Signal.

[9] Okabe K, Hayashi M, Kato K, Okumura M, Fukui R, Honoki K (2013). Lysophosphatidic acid receptor-3 increases tumorigenicity and aggressiveness of rat hepatoma RH7777 cells. Mol Carcinog.

[10] Fukui R, Tanabe E, Kitayoshi M, Yoshikawa K, Fukushima N, Tsujiuchi T (2012). Negative regulation of cell motile and invasive activities by lysophosphatidic acid receptor-3 in colon cancer HCT116 cells. Tumour Biol.

[11] Hayashi M, Okabe K, Yamawaki Y, Teranishi M, Honoki K, Mori T (2011). Loss of lysophosphatidic acid receptor-3 enhances cell migration in rat lung tumor cells. Biochem Biophys Res Commun.

[12] Furui T, LaPushin R, Mao M, Khan H, Watt SR, Watt MA (1999). Overexpression of edg-2/vzg-1 induces apoptosis and anoikis in ovarian cancer cells in a lysophosphatidic acid-independent manner. Clin Cancer Res.

[13] Tsujino M, Fujii M, Okabe K, Mori T, Fukushima N, Tsujiuchi T (2010). Differential expressions and DNA methylation patterns of lysophosphatidic acid receptor genes in human colon cancer cells. Virchows Arch.

[14] Okabe K, Hayashi M, Wakabayash IN, Yamawaki Y, Teranishi M, Fukushima N (2010). Different expressions and DNA methylation patterns of lysophosphatidic acid receptor genes in mouse tumor cells. Pathobiology..

[15] Pan Y, Liu G, Zhou F, Su B, Li Y (2018). DNA methylation profiles in cancer diagnosis and therapeutics. Clin Exp Med.

[16] Jones PA (2012). Functions of DNA methylation: islands, start sites, gene bodies and beyond. Nat Rev Genet.

[17] Sasaki K, Umeda M, Sakai A, Yamazaki S, Tanaka N (2015). Transformation assay in Bhas 42 cells: a model using initiated cells to study mechanisms of carcinogenesis and predict carcinogenic potential of chemicals. J Environ Sci Health C Environ Carcinog Ecotoxicol Rev.

[18] Lee M, Ahn J-H, K.-H. E. (2009). The difference in biological properties between parental and v-ha-ras transformed NIH3T3 cells. Cancer Res Treat.

[19] Yeom H, Hwang SH, Kim H-G, Lee M (2022). Increase in the sensitivity to PLX4720 through inhibition of transcription factor EB-dependent autophagy in BRAF inhibitor-resistant cells. Toxciol Res.

[20] Hwang SH, Han B-I, Lee M (2018). Knockout of ATG5 leads to malignant cell transformation and resistance to Src family kinase inhibitor PP2. J Cell Physiol.

[21] Ahn J-H, Park SN, Yum Y-N, Kim J-Y, Lee M (2008). Comparison of in vitro cell transformation assay using murine fibroblasts and human keratinocytes. Toxicol Sci.

[22] Kajiwara Y, Ajimi S (2003). Verification of the BALB/c 3T3 cell transformation assay after improvement by using an ITES-medium. Toxicol in Vitro.

[23] Geback T, Schulz MM, Koumoutsakos P, Detmar M (2009). TScratch: a novel and simple software tool for automated analysis of monolayer wound healing assays. Biotechniques..

[24] Kim J-H, Ahn J-H, Lee M (2017). Upregulation of microRNA-1246 is associated with BRAF inhibitor resistance in melanoma cells with mutant BRAF. Cancer Res Treat.

[25] Livak KJ, Schmittgen TD (2001). Analysis of relative gene expression data using real-time quantitative PCR and the 2(−Delta Delta C(T)). Method Methods.

[26] Herman JG, Baylin SB (2003). Gene silencing in cancer in association with promoter hypermethylation. N Engl J Med.

[27] Kröger C, Afeyan A, Mraz J, Eaton EN, Reinhardt F, Khodor YL (2019). Acquisition of a hybrid E/M state is essential for tumorigenicity of basal breast cancer cells. Proc Natl Acad Sci U S A.

[28] Ishii I, Contos JJ, Fukushima N, Chun J (2000). Functional comparisons of the lysophosphatidic acid receptors, LP(A1)/VZG-1/EDG-2, LP(A2)/EDG-4, and LP(A3)/EDG-7 in neuronal cell lines using a retrovirus expression system. Mol Pharmacol.

[29] Colhado Rodrigues BL, Lallo MA, Perez EC (2020). The controversial role of autophagy in tumor development: a systematic review. Immunol Investig.

[30] Yang J, Xu J, Han X, Wang H, Zhang Y, Dong J (2018). Lysophosphatidic acid is associated with cardiac dysfunction and hypertrophy by suppressing autophagy via the LPA3/AKT/mTOR pathway. Front Physiol.

[31] Zhang Z, Singh R, Aschner M (2016). Methods for the detection of autophagy in mammalian cells. Curr Protoc Toxicol.

[32] Guo JY, Chen HY, Mathew R, Fan J, Strohecker AM, Karsli-Uzunbas G (2011). Activated Ras requires autophagy to maintain oxidative metabolism and tumorigenesis. Genes Dev.

[33] Su Y, Qian H, Zhang J, Wang S, Shi P, X. P. (2005). The diversity expression of p62 in digestive system cancers. Clin Immunol.

[34] Schmukler E, Kloog Y, Pinkas-Kramarski R (2014). Ras and autophagy in cancer development and therapy. Oncotarget..

[35] Chappell WH, Steelman LS, Long JM, Kempf RC, Abrams SL, Franklin RA (2011). Ras/Raf/MEK/ERK and PI3K/PTEN/Akt/mTOR inhibitors: rationale and importance to inhibiting these pathways in human health. Oncotarget..

[36] Fang X, Gaudette D, Furui T, Mao M, Estrella V, Eder A (2000). Lysophospholipid growth factors in the initiation, progression, metastases, and management of ovarian cancer. Ann N Y Acad Sci.

[37] Fukushima K, Takahashi K, Yamasaki E, Onishi Y, Fukushima N, Honoki K (2017). Lysophosphatidic acid signaling via LPA 1 and LPA 3 regulates cellular functions during tumor progression in pancreatic cancer cells. Exp Cell Res.

[38] Ueda N, Minami K, Ishimoto K, Tsujiuchi T (2020). Effects of lysophosphatidic acid (LPA) receptor-2 (LPA2) and LPA3 on the regulation of chemoresistance to anticancer drug in lung cancer cells. Cell Signal.

[39] Jia W, Tran SK, Ruddick CA, Murph MM (2015). The Src homology 3 binding domain is required for lysophosphatidic acid 3 receptor-mediated cellular viability in melanoma cells. Cancer Lett.

[40] Singh SS, Vats S, Chia AY, Tan TZ, Deng S, Ong MS (2018). Dual role of autophagy in hallmarks of cancer. Oncogene..

[41] Button RW, Roberts SL, Willis TL, Hanemann CO, Luo S (2017). Accumulation of autophagosomes confers cytotoxicity. J Biol Chem.

[42] Perera RM, Stoykova S, Nicolay BN, Ross KN, Fitamant J, Boukhali M (2015). Transcriptional control of autophagy-lysosome function drives pancreatic cancer metabolism. Nature..

[43] Zuckerman V, Sokolov E, Swet JH, Ahrens WA, Showlater V, Iannitti DA (2016). Expression and function of lysophosphatidic acid receptors (LPARs) 1 and 3 in human hepatic cancer progenitor cells. Oncotarget..

[44] Choi JW, Herr DR, Noguchi K, Yung YC, Lee C-W, Mutoh T (2010). LPA receptors: subtypes and biological actions. Annu Rev Pharamcol Toxicol.

[45] Xiang XY, Yang XC, Su J, Kang JS, Wu Y, Xue YN (2016). Inhibition of autophagic flux by ROS promotes apoptosis during DTT-induced ER/oxidative stress in HeLa cells. Oncol Rep.

[46] Luo S, Rubinsztein DC (2010). Apoptosis blocks Beclin 1-dependent autophagosome synthesis: an effect rescued by Bcl-xL. Cell Death Differ.

[47] Wirawan E, Vande Walle L, Kersse K, Cornelis S, Claerhout S, Vanoverberghe I (2010). Caspase-mediated cleavage of Beclin-1 inactivates Beclin-1-induced autophagy and enhances apoptosis by promoting the release of proapoptotic factors from mitochondria. Cell Death Dis.

