# Supplementary material for: Paradoxical downregulation of LPAR3 exerts tumor-promoting activity through autophagy induction in Ras-transformed cells
Source: BMC Cancer. 2022 Sep 10;22:969. doi: 10.1186/s12885-022-10053-0 (PMC9463806; doi:10.1186/s12885-022-10053-0)
Supplement: Supplementary file 1 — Additional file 1: Suppl. Fig. S1. Generation and validation of the knockout of LPAR3 in Bhas 42 cells LPAR3 KO was confirmed by sequencing for genomic editing at the target site. [file 12885_2022_10053_MOESM1_ESM.pdf]

Supplementary Fig. S1 (Hwang et al.)

(Biallelic heterozygous)

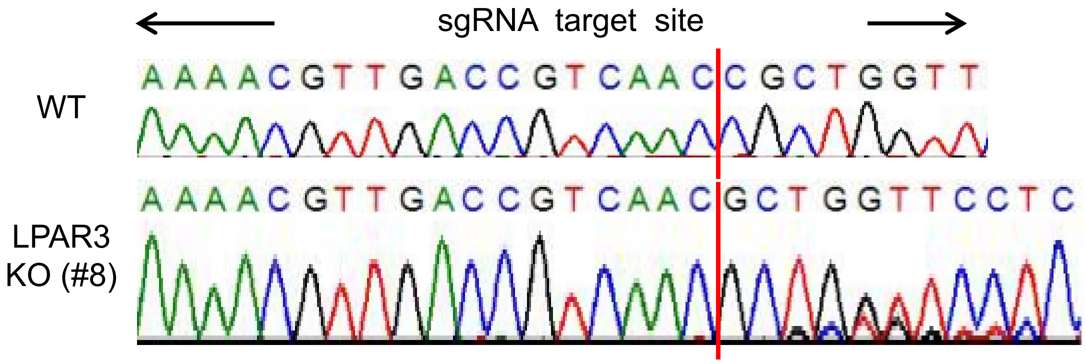

WT aaaacgttgaccgtcaaccgctggtt

#8 - 1 aaaacgttgaccgtcaacgctggtt (1 ntd deletion)

#8 - 2 aaaacgttgaccgtcaacgcgctggtt (1 ntd insertion)

Suppl. Fig. S1. Generation and validation of the knockout of LPAR3 in Bhas 42 cells
LPAR3 KO was confirmed by sequencing for genomic editing at the target site.
