# Supplementary material for: Paradoxical downregulation of LPAR3 exerts tumor-promoting activity through autophagy induction in Ras-transformed cells
Source: BMC Cancer. 2022 Sep 10;22:969. doi: 10.1186/s12885-022-10053-0 (PMC9463806; doi:10.1186/s12885-022-10053-0)
Supplement: Supplementary file 2 — Additional file 2: Supplementary Table S1. Real-time PCR primers sequence for genes with hypermethylated DMRs. [file 12885_2022_10053_MOESM2_ESM.docx]

Supplementary Table S1. Real-time PCR primers sequence for genes with hypermethylated DMRs

| Genes | Forward primer (5’-3’) | Reverse primer (5’-3’) |
| --- | --- | --- |
| WNT9A | GTACCAGTTCCGCTTTGAGC | GATGGCGTAGAGGAAAGCAG |
| EPAS1 | AATCCCTGTTCAAGCCACAC | GCTCCTCCTTCAGTTTGGTG |
| GNG7 | GCATTGAAGCTGGGATCGAA | CACAGTAGCCCATCAGGTCTG |
| AXIN2 | AAGAGAAGCGACCCAGTCAA | TGGATAACTCGCTGTCGTTG |
| LPAR3 | TCGCTTACGTGTTCCTGATG | TTCCACAGCAATAACCAGCA |
| LAMA5 | ATGGCTCCTTGCATACAACC | GAGCCAGCTTCACAGTAGGG |
