# Supplementary material for: Paradoxical downregulation of LPAR3 exerts tumor-promoting activity through autophagy induction in Ras-transformed cells
Source: BMC Cancer. 2022 Sep 10;22:969. doi: 10.1186/s12885-022-10053-0 (PMC9463806; doi:10.1186/s12885-022-10053-0)
Supplement: Supplementary file 3 — Additional file 3: Supplementary Table S2. Real-time PCR primers sequence for genes associated with EMT. [file 12885_2022_10053_MOESM3_ESM.docx]

Supplementary Table S2. Real-time PCR primers sequence for genes associated with EMT

| Genes | Function | Forward primer (5’-3’) | Reverse primer (5’-3’) |
| --- | --- | --- | --- |
| Epcam | Epithelial Cell Adhesion Molecule; A transmembrane glycoprotein mediating Ca^2+^-independent homotypic cell–cell adhesion in epithelia | CCTGAGAGTGAACGGAGAGC | GACACCACCACAATGACAGC |
| Cdh1 | Cadherin 1; Calcium-dependent cell adhesion proteins | ATCCTCTATTCTCATGCCGTGT | AACTCTGGCCTGTTGTCATTCT |
| Esrp1 | Epithelial Splicing Regulatory Protein 1; mRNA splicing factor | GTCAGGAGATGCCTTTATCCAG | CCCCCATTAACACAAAGTTCAT |
| Esrp2 | Epithelial Splicing Regulatory Protein 2; mRNA splicing factor | CTCGAAGCCAGAAGTGGTAAAG | ATGTTGAGCCCTTTGAAGAATC |
| Krt5 | Keratin 5; structural constituent of cytoskeleton | CATTAACAACCTCCGTAGACAGC | CATCCACATCCTTCTTCAACATC |
| Vim | Vimentin; Type III intermediate filament protein | CCAAGTTTGCTGACCTCTCTG | ACCTGTCTCCGGTACTCGTTT |
| Cdh2 | Cadherin 2; Calcium-dependent cell adhesion protein | TATGAGTGGGACAGGAACACTG | GAGTTGGGTTCTGGAGTTTCAC |
| Zeb1 | Zinc Finger E-Box Binding Homeobox 1; Acts as a transcriptional repressor | CACATTAAGTACCGCCATGAGA | ACGTTCAAGCTGGGTTCTGTAT |
| Zeb2 | Zinc Finger E-box-Binding Homeobox 2; R-SMAD-binding protein and acts as a transcriptional corepressor | CAGCATGAACGATACCTGTGTAA | GATGACAAGAGGAGGGCTTTATT |
| Twist1 | Twist Family BHLH Transcription Factor 1; Transcriptional regulator | GTCCCACTAGCAGCGGAG | CACGCCCTGATTCTTGTGAA |
| Twist2 | Twist Family BHLH Transcription Factor 2; Transcriptional regulator | CTGCTCAGCTAGCCGTGTTT | TCCTGGGTGTGGAGCGTTAT |
| Snail1 | Snail Family Transcriptional Repressor 1; RNA polymerase II regulatory region sequence-specific DNA binding | CAACTATAGCGAGCTGCAGGA | ACTTGGGGTACCAGGAGAGAGT |
| Snail2 | Snail Family Transcriptional Repressor 2 | CTCACCTCGGGAGCATACAGC | TGAAGTGTCAGAGGAAGGCGGG |
