# Supplementary figures and images for: Paradoxical downregulation of LPAR3 exerts tumor-promoting activity through autophagy induction in Ras-transformed cells
Source: BMC Cancer. 2022 Sep 10;22:969. doi: 10.1186/s12885-022-10053-0 (PMC9463806; doi:10.1186/s12885-022-10053-0)

Figure 6B

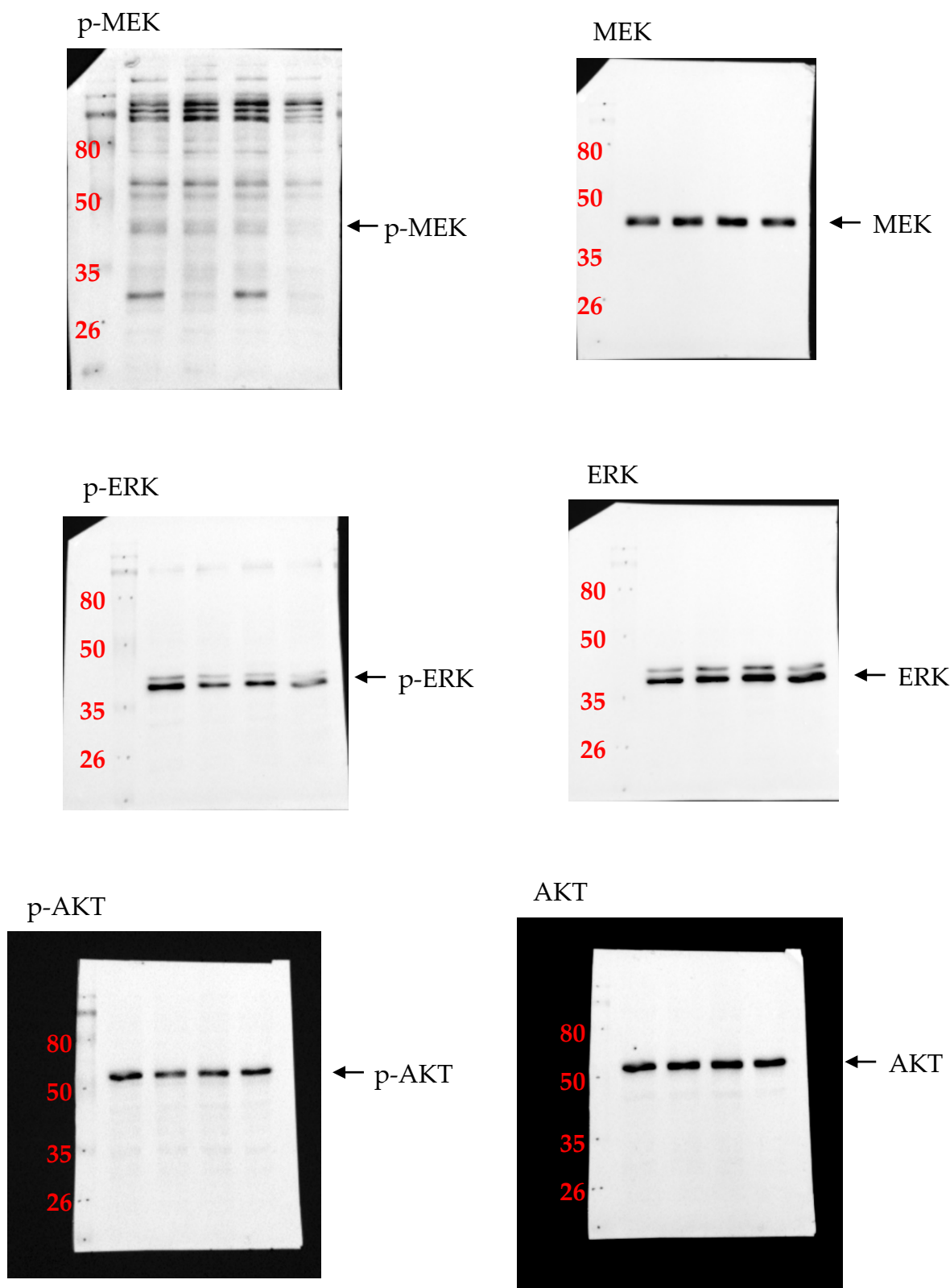

Figure 7A

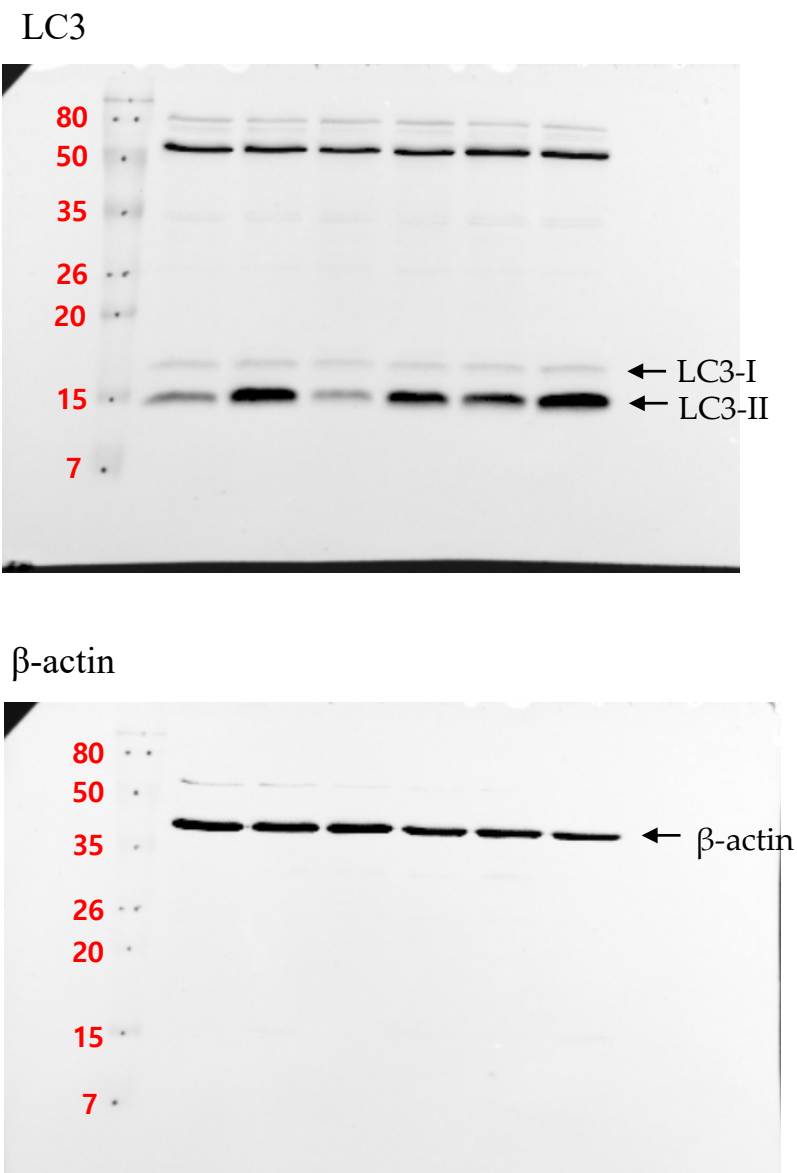

Figure 7B

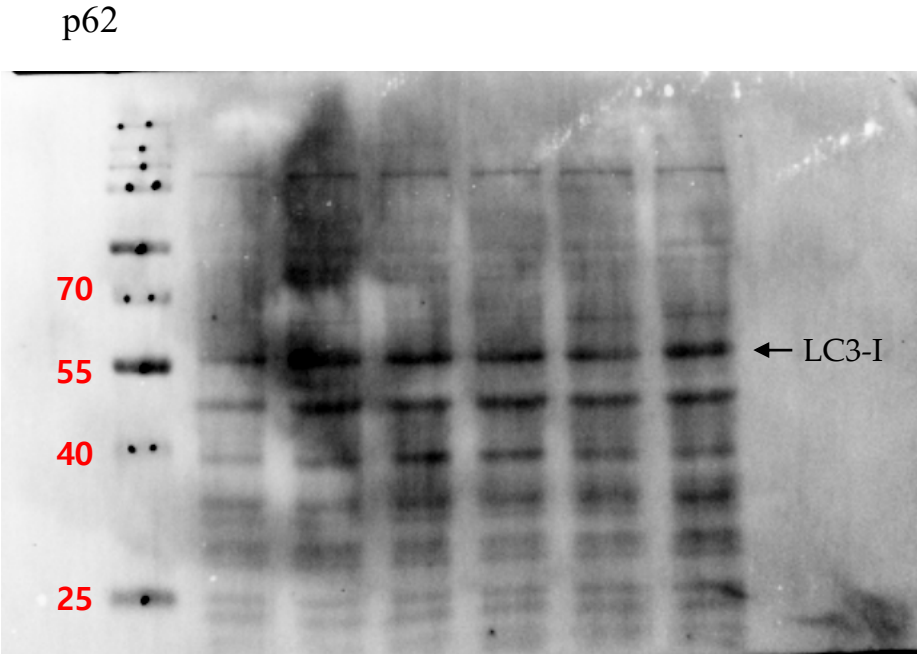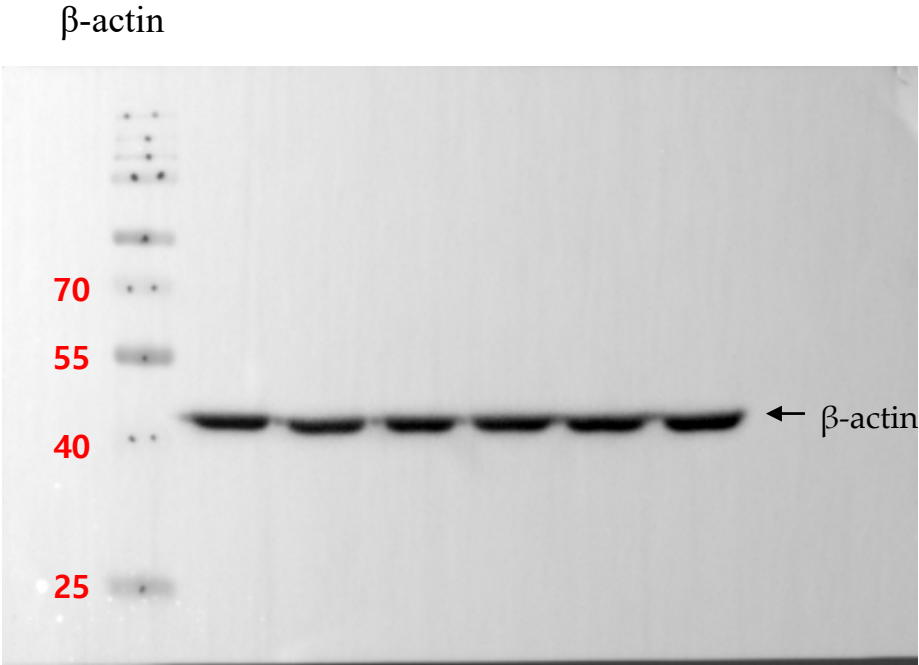

**Figure 7E**

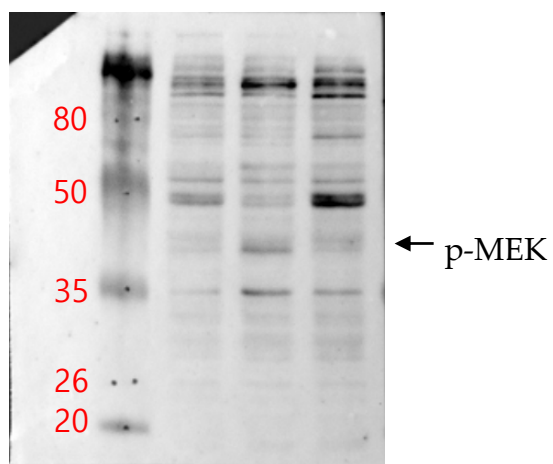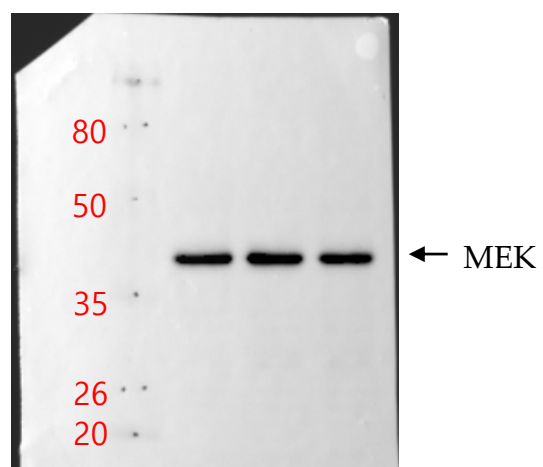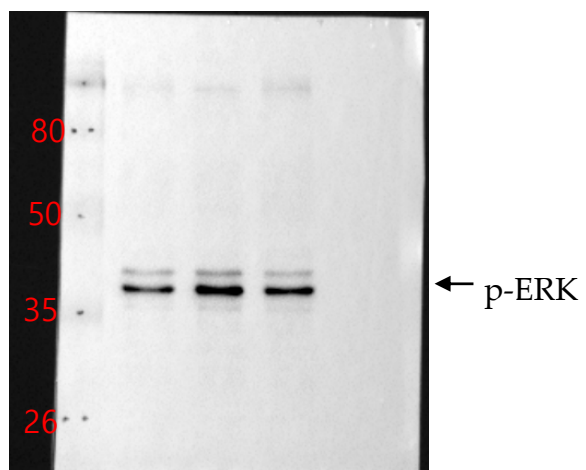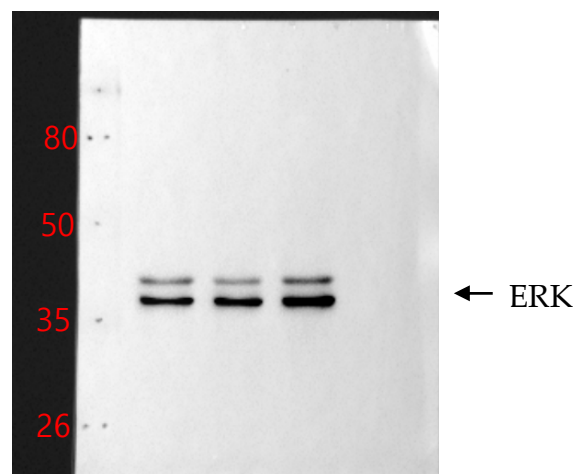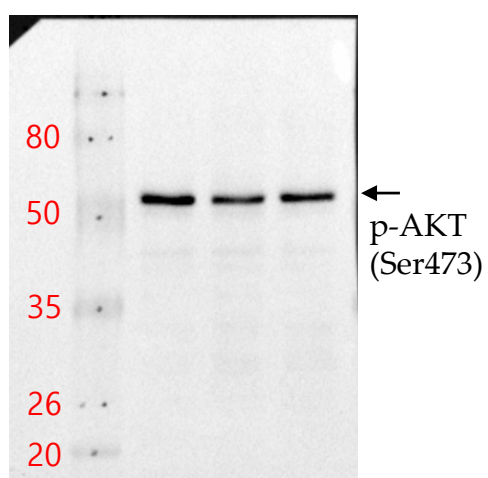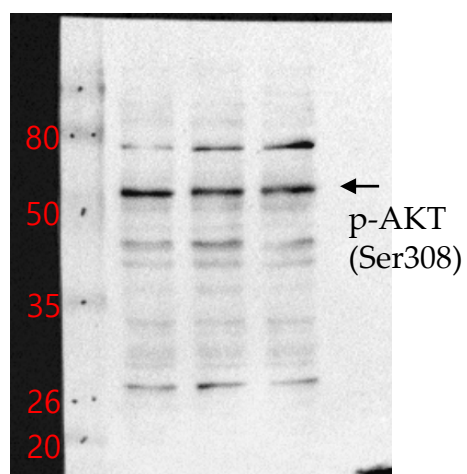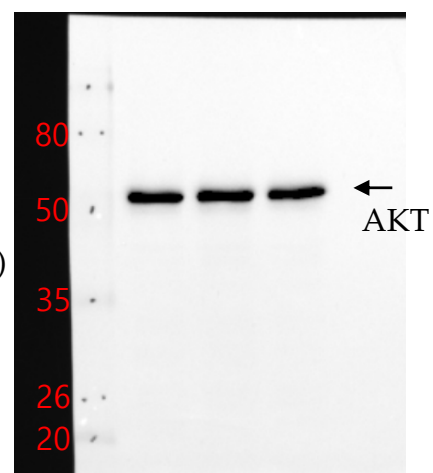

Figure 7E (continued)

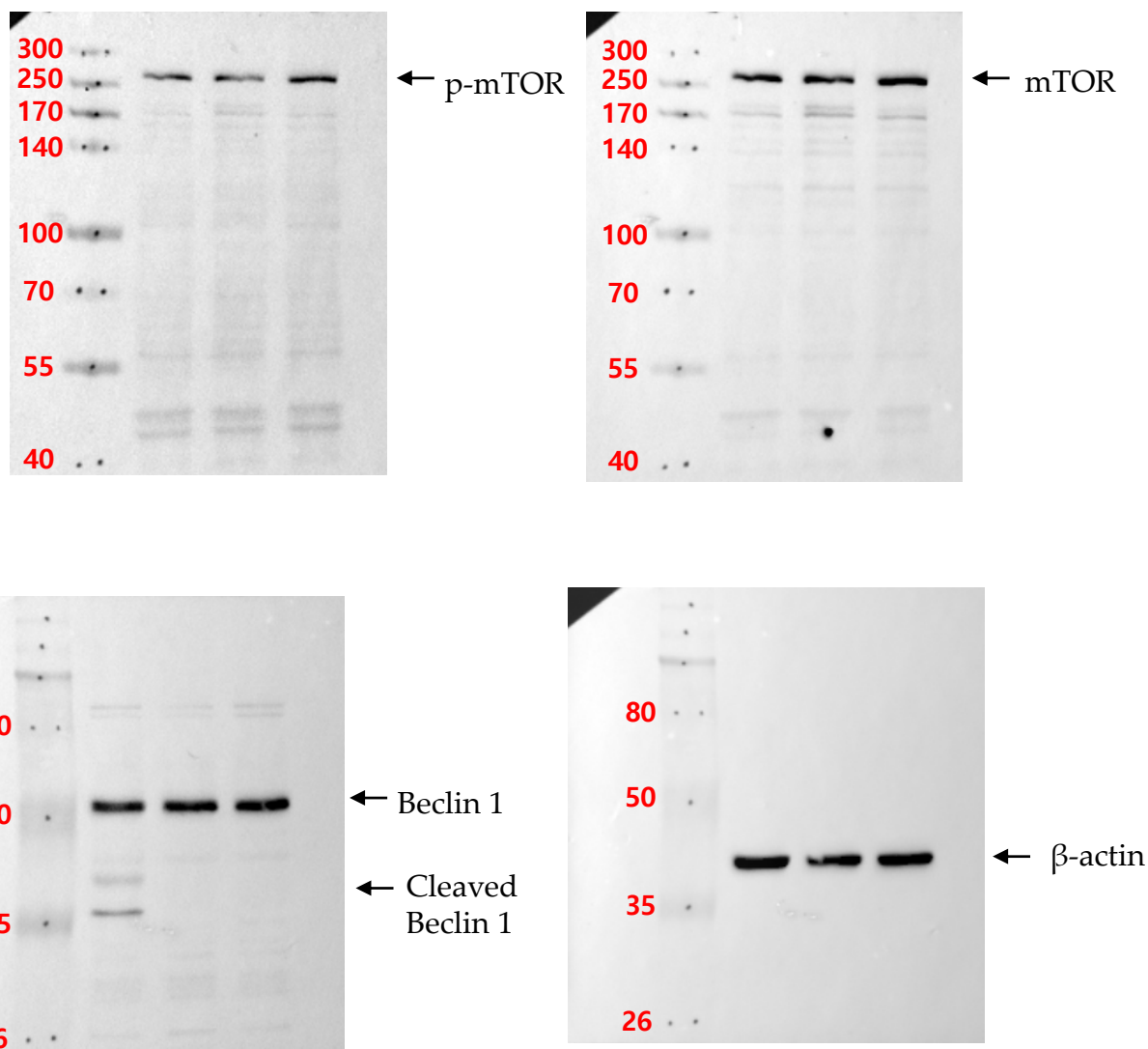

Supplement: Supplementary file 4 — Additional file 4. [file 12885_2022_10053_MOESM4_ESM.pdf]
